# Microbial composition associated with biliary stents in patients undergoing pancreatic resection for cancer

**DOI:** 10.1038/s41522-024-00506-8

**Published:** 2024-03-30

**Authors:** Aitor Blanco-Míguez, Sara Carloni, Cindy Cardenas, Carola Conca Dioguardi, Luca Lambroia, Giovanni Capretti, Gennaro Nappo, Alessandro Fugazza, Antonio Capogreco, Federica Armanini, Francesco Asnicar, Leonard Dubois, Davide Golzato, Paolo Manghi, Federica Pinto, Cristina Scuderi, Erminia Casari, Marco Montorsi, Andrea Anderloni, Maria Rescigno, Alessandro Repici, Alessandro Zerbi, Clelia Peano, Sabrina Tamburini, Roberto Rusconi, Nicola Segata

**Affiliations:** 1 Department CIBIO, University of Trento, Trento, Italy; 2 Department of Biomedical Sciences, Humanitas University, Pieve Emanuele, Italy; 3 IRCCS Humanitas Research Hospital, Rozzano, Italy; 4 Institute of Genetics and Biomedical Research, UoS of Milan, National Research Council, Rozzano, Milan, Italy; 5 Department of Molecular and Translational Medicine, University of Brescia, Brescia, Italy; 6 Department of Pancreatic Surgery, IRCCS Humanitas Research Hospital, Rozzano, Italy; 7 Department of Gastroenterology, IRCCS Humanitas Research Hospital, Rozzano, Italy; 8 Microbiology and Virology Unit, IRCCS Humanitas Research Hospital, Rozzano, Italy; 9 Human Technopole, Milan, Italy; 10 IEO, European Institute of Oncology IRCCS, Milan, Italy

**Keywords:** Applied microbiology, Metagenomics, Biofilms

## Abstract

Malignant bile duct obstruction is typically treated by biliary stenting, which however increases the risk of bacterial infections. Here, we analyzed the microbial content of the biliary stents from 56 patients finding widespread microbial colonization. Seventeen of 36 prevalent stent species are common oral microbiome members, associate with disease conditions when present in the gut, and include dozens of biofilm- and antimicrobial resistance-related genes. This work provides an overview of the microbial communities populating the stents.

## Introduction

Obstructive jaundice is a condition which prevents the normal drainage of bile into the intestines, and it is the most common sign of presentation of pancreatic head or periampullary cancer^1^ for which it is often associated with poor outcomes and decreased survival. Nowadays more than 70% of the patients with biliary obstructive jaundice are treated by biliary stenting in first-line centers receiving the patient under urgent conditions and later referred to specialized high-volume centers for surgery^2^.

Biliary stents are made of either plastic or metal. The major advantage of plastic stents is that they can be removed and replaced if necessary, whereas self-expanding metal stents are permanent but they have the advantage of a larger lumen and longer patency^3^. In recent years, biliary biodegradable stents have also been developed for endoscopic use^4^. However, studies comparing the properties and safety of different types of stents for preoperative biliary drainage are limited and no consensus has been reached on the optimal stent type^5^. More in-depth analyses are thus needed also because it has been suggested that biofilm formation on biliary stents could play a crucial role in the clogging process^6,7^.

The gallbladder has been historically considered an hostile territory for bacteria, mostly due to the antimicrobial properties of bile acids, particularly their detergent effect leading to the dissolution of bacterial membranes^8^. However, recent studies on pigs and human individuals have shown that a healthy gallbladder can harbor diverse microbial taxa, including those from the phyla Firmicutes, Bacteroidetes, Actinobacteria, and Proteobacteria^8,9^. Moreover, previous studies employing traditional cultivation methods have shown an association between biliary stents insertion and a dramatic increase in the colonization of the bile, which was preferentially characterized by species from the duodenal microbiota such as enterococci^10,11^. However, the data available so far, particularly in the case of anaerobic bacteria, are too scarce for a comprehensive description of this phenomenon. In addition, traditional culture methods are not indicative of biofilm growth^12^. We thus studied here the microbial communities present in the stents via cultivation-free shotgun metagenomics^13^.

To investigate the composition of the microbial communities and biofilms colonizing biliary stents, we collected the biliary stents from 56 patients (Supplementary table 1). Study participants were between 32 and 89 years old (avg. 67.30 ± 15.75), and they were carrying the stent for a period between 13 and 330 days (avg. 70.21 ± 73.35). Among these 56 patients, the stents for 17 of them were collected during routine endoscopic procedures and 39 during pancreaticoduodenectomy (PD) surgical procedures. Microbial DNA was extracted from the biofilms grown on the inner surface of the stents and subjected to shotgun metagenomic sequencing (see Methods). Moreover, we further isolated and sequenced 15 different bacterial strains from a random subset of 8 stents (7 metallic and 1 plastic, Supplementary Table 2). After applying a strict quality control preprocessing on the metagenomic reads (Methods), we retained 47 samples with enough sequencing depth (>2 M microbial reads).

**Fig. 1:**
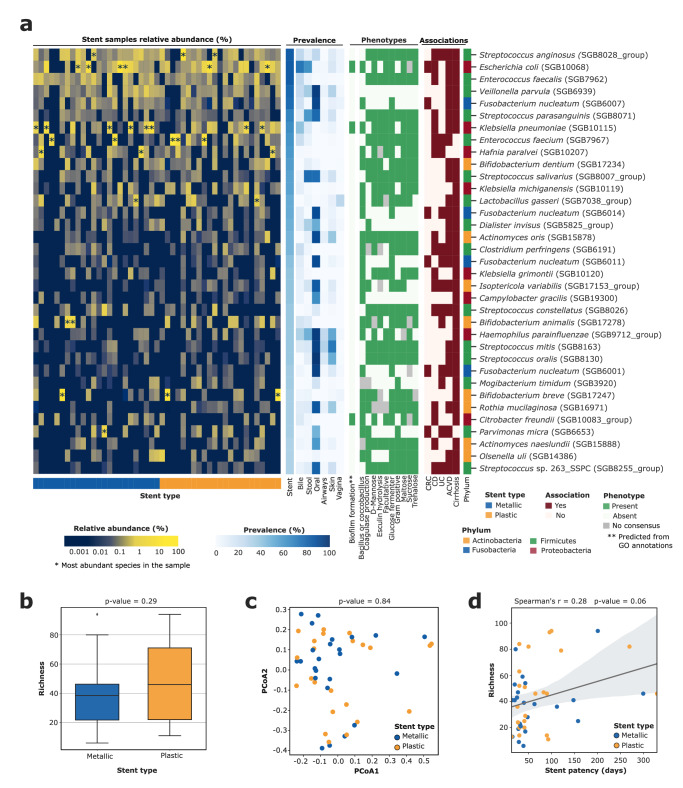
Microbial composition of the biliary stent samples. **a** Most prevalent SGBs in the biliary stents (prevalence >30%). Abundance labeled with asterisks represents the most abundant (dominant) species in the sample. Prevalence in the human body sites was assessed using only samples from healthy donors with the exception of the bile samples (see Methods). Microbial traits were predicted using Traitar on each SGB’s core genes (50% coreness). Traits labeled with two asterisks were selected based on Gene Ontology (GO) annotations of the SGB’s core genes (50% coreness). Associations with diseases were defined by FDR *p* < 0.2 and effect size >0.2. **b** Microbial richness by stent material (Mann-Whitney U test *p* = 0.29). **c** Principal coordinate analysis based on the Bray-Curtis dissimilarity finds no detectable differences between the microbial composition of metallic versus plastic stents (PERMANOVA *p* = 0.84). **d** Microbial richness by stent patency (Spearman’s *r* = 0.28, *p*-value = 0.06). CRC Colorectal cancer, CD Crohn disease, UC Ulcerative colitis, ACVD Atherosclerotic cardiovascular disease. Box plots in **b** show the median (center), 25th/75th percentile (lower/upper hinges), 1.5× interquartile range (whiskers), and outliers (points).

We applied MetaPhlAn 4^14^ over the full set of quality-controlled stent samples, and identified a total of 364 species-level genome bins (SGBs, avg. 42 ± 25 per sample)^15^ present in at least one stent. Twenty-eight of these SGBs represent species without taxonomically characterized representatives (“unknown” SGBs, i.e. uSGBs, Supplementary Figure 1, Supplementary table 3). The microbial communities colonizing the stents were shown to be very different between patients, with few dominant bacteria in each sample that are, however, different between samples (Fig. 1a). In fact, only three species were detected in more than 80% of the samples (prevalence = 83.0%, Fig. 1a): *Streptococcus anginosus* (SGB8028 group, avg. relative abundance = 5.7% ± 11.9%), *Escherichia coli* (SGB10068, avg. rel. abundance = 12.8% ± 18.6%) and *Enterococcus faecalis* (SGB7962, avg. rel. abundance = 3.4% ± 5.0%). A larger set of 36 SGBs had >30% prevalence (Fig. 1a) and all belonged to SGBs with cultured representatives (i.e. kSGBs), suggesting the artificial stent environment tends to select for human microbiome members adaptable to more diverse environments with relatively well-established cultivation conditions. None of these 36 prevalent bacterial species were previously identified as common contaminants during laboratory procedures^16^. These stent-associated species were typically facultative anaerobic (50%), gram positive bacteria (58.3%) and able to ferment simple sugars such as D-Mannose (61.1%), Glucose (83.3%), Maltose (69.4%), Sucrose (61.1%), and Trehalose 61.1%, Fig. 1a).

We then tried to investigate the potential body site of origin for stent-colonizing bacteria. *E. coli* (SGB10068), *Klebsiella pneumoniae* (SGB10115), and several lactic acid bacteria (LAB) were commonly the dominant species of the stents (22 out of 47 samples), but only the first two were found with a similar prevalence when assessed samples from human bile^9,17,18^ (Fig. 1a, Supplementary Table 4; see Methods). Instead, almost half of the prevalent species (>30% prevalence) in the stents (17 out of 36) are common members of the human oral microbiome (with a prevalence above 50% in oral samples from healthy individuals in available studies^14^), and, when present in the gut, they are predominantly associated with different diseases such as colorectal cancer (CRC), inflammatory bowel disease (IBD), atherosclerotic cardiovascular disease (ACVD) and cirrhosis (FDR < 0.2, Fig. 1a, Methods). However, none of the species dominating the stents were also oral-associated species (reference prevalence in oral samples below 50%). Overall, the stent-associated microbes have a much lower diversity than the commensal microbiome (avg. SGB richness of the stents = 42 ± 25) of the gastrointestinal tract and the stent environment is selective for a reduced set of mostly oral bacteria that can dominate this artificial tool.

The biliary stents considered in this study were either metallic—i.e., made of braided metal alloy of nickel and titanium(Nitinol), with a silicone polymer lining of its entire length—or plastic—i.e., made of polyethylene—and we tested whether the two different types of stents were colonized by different microbial communities. We did not find any statistically significant differences when assessing alpha diversity based on SGB richness (Fig. 1b, Mann-Whitney U test *p* = 0.29), Shannon diversity (Supplementary Fig. 2a, b, *p* = 0.83) or Simpson diversity (*p* = 0.99) as well as intra-type beta diversity (Fig. 1c, PERMANOVA on Bray-Curtis distances *p* = 0.84). Likewise, no statistical associations were found between SGBs and the different stent materials (FDR > 0.2). We did find a significant SGB richness increase when comparing samples retrieved from endoscopy to those retrieved during the pancreatic surgery (Supplementary Figure 2c, Mann-Whitney U test *p* = 0.018). However, this increase seems to be directly related to the significantly smaller number of reads in the latest ones (Supplementary Fig. 2d, Mann-Whitney U test *p* = 0.0059) due to a significantly higher contamination from human DNA during the stent extraction (Supplementary Fig. 2e, Mann-Whitney U test *p* = 4.19e-5). No significant differences were either found when comparing the gender, complications after the surgery (measured with the Clavien-Dindo Classification) or the physical status before the stent removal (measured with the American Society of Anesthesiologists (ASA) Physical Status Classification System) of the patients (Supplementary Fig. 2g–i). However, since most of the Clavien-Dindo and ASA classifications are spanned by a very limited sample set, most of the comparisons are not statistically relevant. We found, however, a small and borderline significant correlation between the microbial richness of the samples and the stent patency (Fig. 1d, Spearman’s r = 0.28, *p*-value = 0.06). Altogether, these results show that the microbial composition of the biliary stents is not particularly affected by the stent material or the extraction procedure, nor by any of the considered characteristics of the patients.

**Fig. 2:**
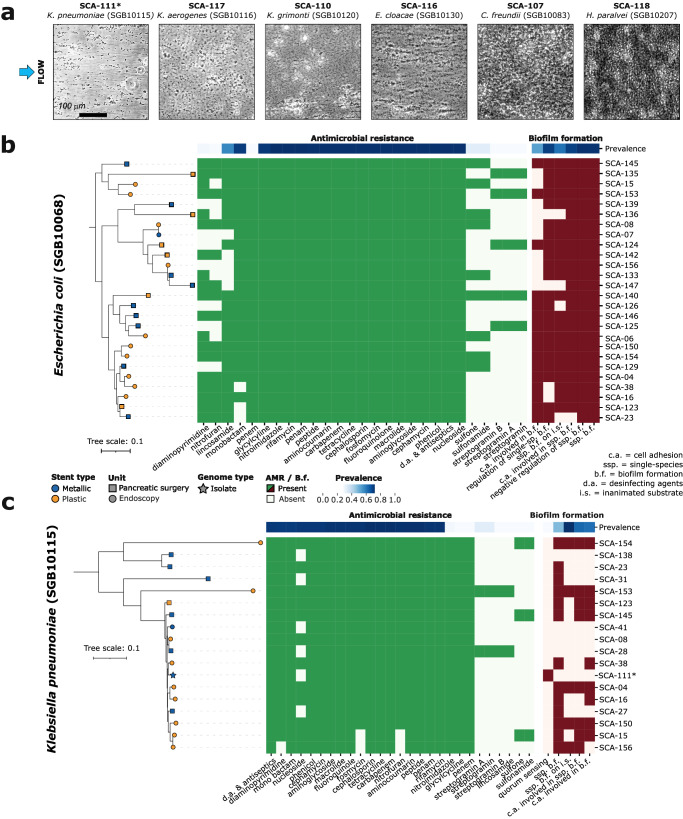
Experimental and strain-level and characterization of the microbial species colonizing the biliary stents. **a** Biofilm formation under continuous flow after 6 h of growth for 6 different microbial isolates retrieved from the stents. Phase-contrast images taken at the bottom surface of a microfluidic channel are shown. **b**, **c** Phylogenetic reconstruction, antimicrobial resistance (AMR) and biofilm formation profiles of the two most abundant and prevalent species in the stents, **b** *Escherichia coli* and **c** *Klebsiella pneumoniae*, from patient samples. Phylogenetic trees were built using both metagenomic samples and isolated genomes with StrainPhlAn 4. Samples from metallic stents are colored in blue and those from plastic ones in orange. Samples retrieved during the pancreatic surgery are represented by squares and samples retrieved from endoscopy as circles. Isolated sequences from the stents are represented as stars. Blue heatmap represents the prevalence of each AMR or biofilm formation-related gene in the species pangenome.

Bacterial strains we cultivated from the stents were experimentally studied for their ability to develop biofilms under clinically relevant conditions using microfluidics (Fig. 2a). We also performed a phylogenetic and functional potential analysis of the two most dominant species, *Escherichia coli* (SGB10068) and *Klebsiella pneumoniae* (SGB10115), combining the strain-level genetic analysis results of StrainPhlAn 4^14^ and the functional potential profiles retrieved by PanPhlAn 3^19^. We found that all reconstructed *E. coli* strains were functionally predicted as biofilm formers, as expected from the high prevalence of biofilm-related genes in the pangenome of the stent-associated strains (Fig. 2b). We also annotated a large collection of AMR-related genes in our *E. coli* strains. While most of these resistances were shared by most of the *E. coli* strains coming from other sources (present in more than 90% of the species’ strains in public repositories), we found some resistances that were specific to our samples (Fig. 2b). Resistance to diaminopyrimidine, nitrofuran, penem, sulfone, sulfonamide and streptogramin were found specific from the stent samples (with a prevalence in the species’ pangenome below 20%), being the resistance to penem present in all our strains (*n* = 26). Phylogenetically, *E. coli* strains colonizing the stents were quite heterogeneous, and we found only two potential strain-sharing events between samples from different patients (Fig. 2b, see Methods). As for the whole microbiome, we did not find any specific strain associated with the type of material of the stents. On the other hand, biofilm formation is not as prevalent in our *K. pneumoniae* strains, but we still predicted this specific phenotype in more than half of them (Fig. 2c). Similarly to *E. coli*, AMR genes are quite abundant also in *K. pneumoniae*, with resistances to nitroimidazole, glycylcycline and penem almost absent in the species’ pangenome (in less than 20% of the species’ strains) but highly prevalent in the stent’s strains (Fig. 2c). Again we did not find any phylogenetic association with the stent material while the phylogenetic diversity of the *K. pneumoniae* strains retrieved from the stents was limited (Fig. 2c).

Altogether, our study of the microbial communities associated with biliary stents constitutes a proof-of-concept for the impact of metagenomic approaches in this clinical setting. We show that these communities are quite rich and diverse between patients, but they are not particularly affected by the stent material, extraction procedure or phenotypical characteristics of the patients. Moreover, strain-level functional and phylogenetic analyses of the most dominant species show a particularly high prevalence of biofilm- and antimicrobial resistance-related genes in most of the stent strains. While almost half of the prevalent species are common members of the human oral microbiome, further work is still needed to understand the origin of these microorganisms, the route from which they populate the stents, and their role in the clinical outcome of the patients.

## Methods

### Stents sample collection and processing

Internal biliary stents (either made of plastic or metal) were retrieved from both the Digestive Endoscopy Unit and the Pancreatic Surgery Unit at the Humanitas Clinical and Research Center (Rozzano, Milan, Italy). Seventeen biliary stents were collected during routine endoscopic procedures and stored in sterile containers at −80 °C. A total of 39 biliary stents were retrieved during pancreaticoduodenectomy (PD) surgical procedures and stored in sterile containers at −80 °C. Our team adhered to established aseptic techniques to minimize the introduction of external microbial contaminants throughout the entire process. All stents were collected from patients that did not have any antibiotic prophylaxis or chemotherapy. Stent patency times ranged between 5 and 330 days. This study has been approved by the Ethics Committee of IRCCS Humanitas Research Hospital (no. 11/20).

### Isolation of bacterial strains

Sixteen bacterial strains (including *K. aerogenes, C. freundii, C. braakii, K. michiganensis, K. grimontii, K. pneumoniae, E. faecalis, P. aeruginosa, E. cloacae, E. ludwigii, H. paralvei, S. maltophilia, E. coli*) from 8 different stents were identified by the Microbiology Unit at the Humanitas Clinical and Research Center. Retrieved stents were placed in a sterile container with physiological solution. To disrupt the biofilm on the inner surface of the stent, the specimen was vortexed for 30 s and subsequently exposed to low-frequency (40 kHz) ultrasound for 15 min. Thereafter, the container was vortexed again for 30 s, as previously described. Aliquots of 0.1 and 0.01 mL of the sonication fluid were plated on nonselective media and incubated at 37 °C for 48–96 h under aerobic and anaerobic conditions. Isolated microorganisms were then counted and identified at the species level using matrix-assisted laser desorption/ionization-time of flight (MALDI-TOF) mass spectrometry.

### Biofilm phenotyping of the isolates

A selection of the bacterial isolates associated with biliary stents (selected based on their robust growth when cultivated) were grown in a general-purpose culture medium (Tryptone Broth) and then injected into microfluidic channels (height = 106 μm, width = 800 μm, length = 12 mm) previously sealed via plasma bonding onto a microscope glass slide. The ability of these pathogens to attach to the bottom surface of the channels and to develop biofilms was monitored over time under physiologically relevant conditions (i.e., at 37 °C), in the presence or absence of fluid flow (flow rate 2 μl/min), using an inverted fully-automated microscope (DMi8, Leica) for 12 h. Phase-contrast images were taken every 5 min in three different positions of the same microfluidic channel.

### DNA extraction, sequencing, and preprocessing

Biliary stents were scraped to separate the biofilm matrix from the stent structure. The biofilm matrix was homogenized in 550 μl of Cell Suspension Solution and DNA was subsequently extracted using a GNOME DNA isolation kit MP Biomedicals™ 112010600 following a published protocol^20^. Sequencing libraries were prepared using the Nextera DNA Flex Library Preparation Kit (Illumina), following the manufacturer’s guidelines. Sequencing was performed on the Illumina NovaSeq 6000 platform following manufacturer’s protocols. Sequenced samples were pre-processed using a pipeline described in https://github.com/SegataLab/preprocessing Shortly, metagenomic reads of low quality (quality score <Q20), fragmented short reads (<75 bp), and reads with more than 2 ambiguous nucleotides were removed using Trim Galore version 0.6.6^21^. Contaminant and host DNA was identified using Bowtie2 version 2.3.4.3^22^ with the “--*sensitive-local*” parameter, allowing confident removal of the phiX 174 Illumina spike-in and human-associated reads (hg19 human genome release). Metagenomic samples with less than 2 million microbial reads after the preprocessing were discarded (Supplementary Table 1).

### Isolate genome assembly

Isolate sequences were assembled using SPAdes version 3.15.2^23^ with parameters “-k 21,33,55,77,99,127 --careful”. Contigs were further analyzed using MetaBAT2 version 2.12.1^24^ to remove contigs originating from potential DNA contamination. Completeness and contamination of the assembled isolates were checked using CheckM version 1.1.3^25^.

### Isolate genome annotation

Open reading frames were detected and annotated on all genomes using Prokka version 1.14^26^. Coding sequences (CDS) were then assigned to a UniRef90 cluster^27^ by performing a DIAMOND search (version 0.9.24)^28^ of the CDS against the UniRef90 database (version 201901) and assigning a UniRef90 ID if the mean sequence identity to the centroid sequence was over 90% and if it covered more than 80% of the centroid sequence.

### Metagenomic assembly

Metagenomic samples were assembled using MEGAHIT version 1.1.1^29^ with default parameters. Contigs longer than 1500 nucleotides were binned into metagenome-assembled genomes (MAGs) using MetaBAT2 version 2.12.1^24^. MAGs were quality controlled using checkM version 1.1.3^25^ and genomes estimated to be medium-to-high-quality according to genomic completeness >50% and genomic contamination <5% were kept.

### SGB and strain-level metagenomic profiling

SGB-level metagenomic profiling was performed using MetaPhlAn 4 with the Jan21 markers database using default parameters^14^. Alpha and beta diversity metrics were calculated on the SGB-level taxonomic profiles using the python “skbio.diversity” package version 0.5.7. Beta diversity was calculated from the Bray-Curtis distances based on the arcsin square root-transformed relative abundances. Strain-level phylogenetic analysis was performed using StrainPhlAn 4^14^ with parameters “*–sample_with_n_markers 66 –marker_in_n_samples 66 –mutation_rates*”. The same strains were defined using a cutoff of 0.01 normalized genetic distance (normalized by the total branch length) calculated using the branch length of the tree as previously described^30^. Strain-level functional analysis was performed using PanPhlAn 3^19^ with default parameters.

### Prevalence of the SGBs in the human body

Prevalence of the SGBs in the human body was retrieved from the Blanco-Miguez et al. study^14^. Specifically, prevalences in the airways, gastrointestinal tract, oral, skin, and vagina using only samples from healthy individuals were retrieved. For the prevalences in the bile, the SGB prevalences were assessed using 49 metagenomic samples from 3 public studies^9,17,18^.

### Association with the panel of diseases

We queried the cMD 3 repository^31^ for all stool microbiome samples from adult individuals with available age, sex, and BMI, from case-control settings with at least 10 diseased and 10 healthy participants. In total, we analyzed 12 diseases: 2 nutritional or metabolic diseases (type-2-diabetes, T2D, and atherosclerotic-cardiovascular disease, ACVD, 477 and 305 samples), one psychological pathology (*N* = 171, schizophrenia), a gastrointestinal-tract disease having a tumoral character (colorectal cancer, CRC, *N* = 1300), two gastrointestinal tract autoimmune diseases and a autoimmune non-gastrointestinal tract disease (Crohn’s diseases, CD, ulcerative colitis, UC, asthma, *N* = 309, *N* = 346, and *N* = 200 samples); one multisystem inflammatory disease (Behcet-disease, BD, *N* = 65); one liver disease (cirrhosis, *N* = 237); Soil-Transmitted-Helminths, STH (*N* = 159); one partially uncovered pathology (ref.^32^, myalgic encephalomyelitis or chronic fatigue syndrome ME/CFS, *N* = 100); a partially uncovered etiology disease which involves the brain tough not considered a nervous system disease (migraine, *N* = 226). Species relative abundances were centered log-ratio transformed after imputation of zeros by a multiplication replacement strategy (skbio python library, ver. 0.5.6). In total we analyzed 3,462 individual’s microbiomes (1619 diseased participants and 1843 matched controls). Diseases present with multiple datasets (CRC, CD, UC, and T2D) were synthesized in a random effect-meta-analysis in which the standardized mean differences were extracted by linear models adjusted by sex, age, BMI, and sample’s depth. Diseases present with a single dataset were analyzed via a similar linear model. The resulting four meta-analyses and eight coefficients were synthesized in a random-effects meta-analysis. The whole procedure is available at https://github.com/waldronlab/curatedMetagenomicDataAnalyses.

### Phenotypic traits prediction

Phenotypic traits were predicted for all detected SGBs using Traitar (version 1.1.12)^33^ on the 50% core genes (genes present in 50% of genomes available in the SGB database, ref.^14^). Only annotations for which the phypat and the phypat+PGL classifiers annotations matched were considered. Additionally, the biofilm formation phenotype was predicted by annotating the 50% core genes against the Gene Ontology (GO) database^34^ using the GO terms assignment to UniRef90 IDs from the HUMAnN 3 database^19^.

### Antimicrobial resistance and biofilm formation annotations

Antimicrobial resistance (AMR) and biofilm formation profiles were predicted using the UniRef90 (version 201901) annotations retrieved from the PanPhlAn profiles and the isolate genomes annotations. Specifically, genes from the CARD database (version 3.2.1)^35^ were assigned to a UniRef90 ID after performing a DIAMOND search (version 0.9.24)^28^ against the UniRef90 database (version 201901) if the mean sequence identity to the centroid sequence was over 90% and if it covered more than 80% of the centroid sequence. GO terms assignment to UniRef90 IDs was retrieved from the HUMAnN 3 database^19^. Using the UniRef90 assignment to CARD/GO annotations, and the PanPhlAn profiles and the isolate genomes annotations, the AMR and biofilm formation profiles were retrieved. The prevalence of the AMR and biofilm formation-related UniRef90 clusters in each SGBs were assessed using the SGB genome catalog from the Blanco-Míguez et al.^14^ study.

### Reporting summary

Further information on research design is available in the Nature Research Reporting Summary linked to this article.

### Supplementary information

Supplementary Information

Reporting Summary

Supplementary Tables

## Data Availability

All metagenomes have been deposited and are available at the NCBI Sequence Read Archive (PRJNA984000).
