# Supplementary material for: Microbial composition associated with biliary stents in patients undergoing pancreatic resection for cancer
Source: NPJ Biofilms Microbiomes. 2024 Mar 30;10:35. doi: 10.1038/s41522-024-00506-8 (PMC10981703; doi:10.1038/s41522-024-00506-8)
Supplement: Supplementary file 1 — Supplementary Information [file 41522_2024_506_MOESM1_ESM.pdf]

Supplementary Information

Supplementary Figures

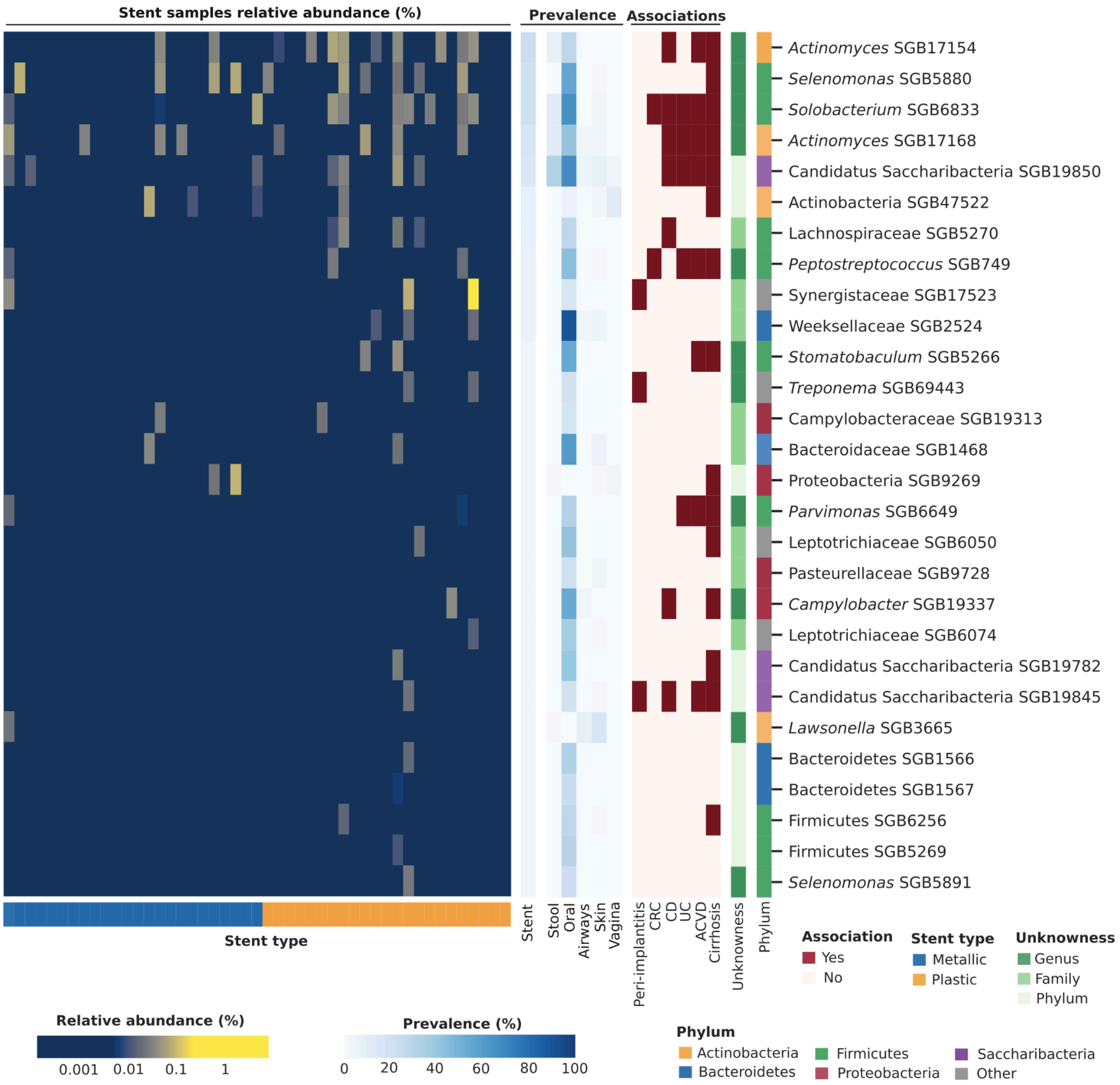

Supplementary Figure 1: Unknown microbial species present in the stent samples. Prevalence in the human body sites was assessed using only samples from healthy donors. Association with diseases was defined by FDR < 0.2 and Effect size >= 0.2. CRC = Colorectal cancer, CD = Crohn disease, UC = Ulcerative colitis, ACVD = Atherosclerotic cardiovascular disease.

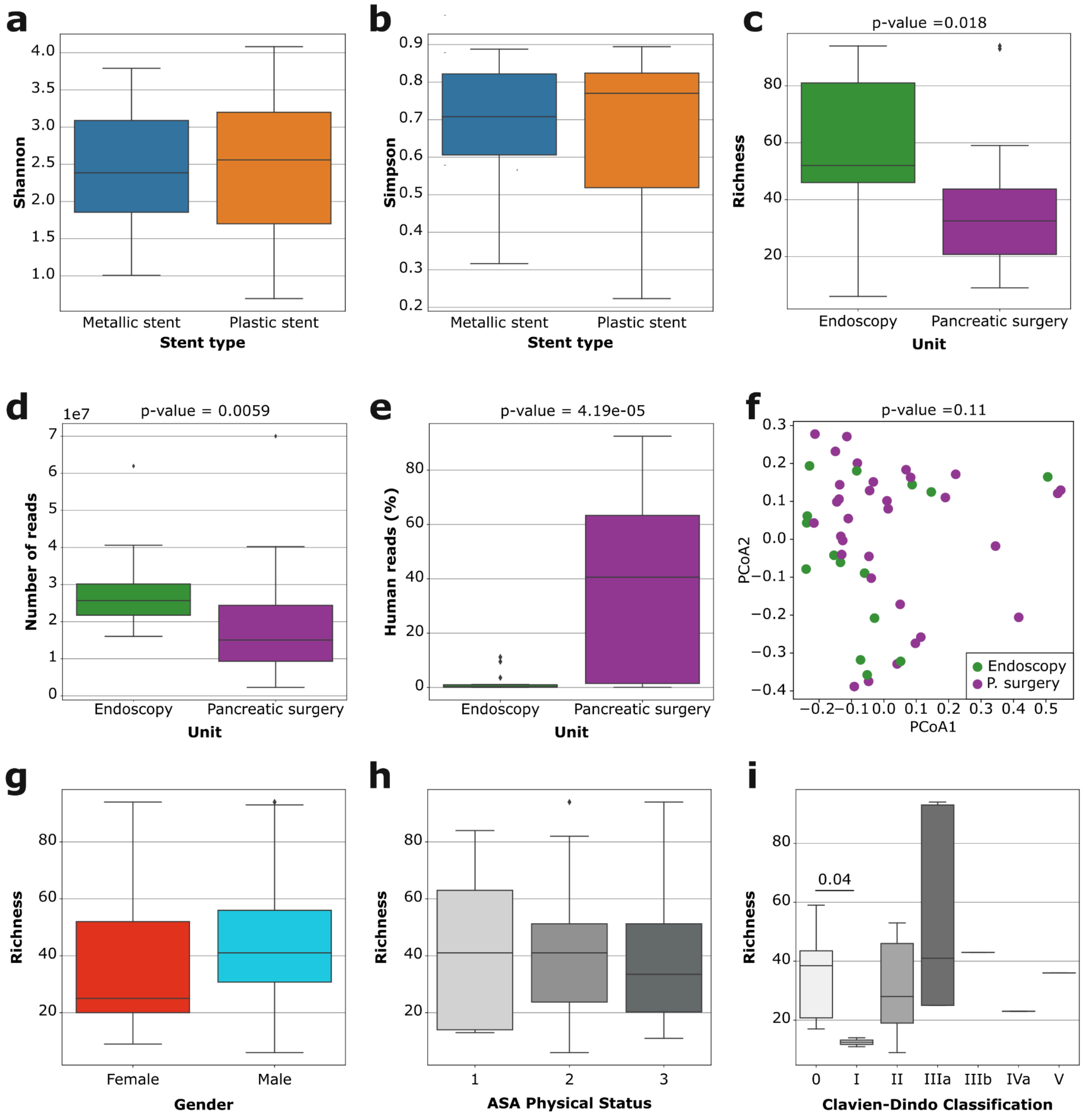

Supplementary Figure 2: Microbial diversity differences when assessing the stents material, retrieval procedure and patient metadata. (a) Shannon diversity difference between samples from different stent materials (Mann-Whitney U test = 0.83). **(b)** Simpson diversity difference between samples from different stent materials (Mann-Whitney U test = 0.99). **(c)** SGB richness difference between samples retrieved by endoscopy and those retrieved during the pancreatic surgery (Mann-Whitney U test = 0.018). **(d)** Difference in the number of reads between samples retrieved by endoscopy and those retrieved during the pancreatic surgery (Mann-Whitney U test = 0.0059). **(e)** Difference in the percentage of human reads contamination in the between the samples retrieved by endoscopy and those retrieved during the pancreatic surgery (Mann-Whitney U test = 4.19e-5). **(f)** Beta diversity based on Bray-Curtis distances between samples retrieved by endoscopy and those retrieved during the pancreatic surgery (PERMANOVA = 0.11). **(b)** SGB richness difference between samples from different genders (Mann-Whitney U test = 0.43). **(h)** SGB richness difference between samples from patients with different physical status before the stent removal (measured with the American Society of Anesthesiologists (ASA) Physical Status Classification System). **(i)** SGB richness difference between samples from patients suffering with different types of complications after the surgery (measured with the Clavien-Dindo Classification). Only significant differences are shown (Mann-Whitney U test p-value < 0.05). Box plots in **a-e** and **g-i** show the median (center), 25th/75th percentile (lower/upper hinges), 1.5× interquartile range (whiskers) and outliers (points).

Supplementary Tables

Supplementary table 1. Metadata associated with the stent samples.

Supplementary table 2. Metadata associated with the isolates from the bile cultures.

Supplementary table 3. MetaPhlAn 4 SGB-level taxonomic profiling of the stent samples.

Supplementary table 4. MetaPhlAn 4 SGB-level taxonomic profiling of the publicly available bile samples.
